# Neurophysiologic Correlates of Post-stroke Mood and Emotional Control

**DOI:** 10.3389/fnhum.2016.00428

**Published:** 2016-08-30

**Authors:** Deniz Doruk, Marcel Simis, Marta Imamura, André R. Brunoni, Leon Morales-Quezada, Renato Anghinah, Felipe Fregni, Linamara R. Battistella

**Affiliations:** ^1^Spaulding Neuromodulation Center, Spaulding Rehabilitation Hospital, Harvard Medical SchoolBoston, MA, USA; ^2^Physical and Rehabilitation Medicine Institute of the University of São Paulo, Medical School General HospitalSão Paulo, Brazil; ^3^Service of Interdisciplinary Neuromodulation, Laboratory of Neurosciences (LIM-27), Department and Institute of Psychiatry, University of São PauloSão Paulo, Brazil

**Keywords:** chronic stroke, qEEG, emotional disturbance, power asymmetry, inter-hemispheric connectivity

## Abstract

**Objective:** Emotional disturbance is a common complication of stroke significantly affecting functional recovery and quality of life. Identifying relevant neurophysiologic markers associated with post-stroke emotional disturbance may lead to a better understanding of this disabling condition, guiding the diagnosis, development of new interventions and the assessments of treatment response.

**Methods:** Thirty-five subjects with chronic stroke were enrolled in this study. The emotion sub-domain of Stroke Impact Scale (SIS-Emotion) was used to assess post-stroke mood and emotional control. The relation between SIS-Emotion and neurophysiologic measures was assessed by using covariance mapping and univariate linear regression. Multivariate analyses were conducted to identify and adjust for potential confounders. Neurophysiologic measures included power asymmetry and coherence assessed by electroencephalography (EEG); and motor threshold, intracortical inhibition (ICI) and intracortical facilitation (ICF) measured by transcranial magnetic stimulation (TMS).

**Results:** Lower scores on SIS-Emotion was associated with (1) frontal EEG power asymmetry in alpha and beta bands, (2) central EEG power asymmetry in alpha and theta bands, and (3) lower inter-hemispheric coherence over frontal and central areas in alpha band. SIS-Emotion also correlated with higher ICF and MT in the unlesioned hemisphere as measured by TMS.

**Conclusions:** To our knowledge, this is the first study using EEG and TMS to index neurophysiologic changes associated with post-stroke mood and emotional control. Our results suggest that inter-hemispheric imbalance measured by EEG power and coherence, as well as an increased ICF in the unlesioned hemisphere measured by TMS might be relevant markers associated with post-stroke mood and emotional control which can guide future studies investigating new diagnostic and treatment modalities in stroke rehabilitation.

## Introduction

Emotional disturbance is a common complication of stroke (Annoni et al., 2006). About 30% of stroke survivors develop anxiety and depressive symptoms critically affecting functional recovery (Parikh et al., 1990; Hackett and Anderson, 2005) and quality of life (Robinson, 1997; Jonsson et al., 2005). Moreover, a significant number of patients remain undetected and therefore untreated due to difficulties in diagnosis (Dafer et al., 2008; El Husseini et al., 2012; Ayerbe et al., 2013). Investigation of neurophysiological markers associated with post-stroke mood and emotional control could have important implications in the development of new interventions as well as the assessment of current diagnostic and therapeutic modalities in stroke rehabilitation. For example, neurophysiologically guided interventions, such as EEG biofeedback entrainment, has already been shown to be effective in stroke patients with physical and cognitive impairments (Nelson, 2007). Similarly in depression, qEEG has been used to detect inter-hemispheric imbalance in cortical activity that has lead to the application of new therapeutic approaches such as TMS (transcranial magnetic stimulation) and tDCS (transcranial direct current stimulation; Rosenfeld et al., 1996; Linden, 2014).

The exact causes of post-stroke emotional disturbance (PS-ED) are still unknown. Different mechanisms including direct effects of ischemia to mood regulating neural networks (Starkstein et al., 1988; Beblo et al., 1999) and a psychosocial (Gainotti et al., 1999) model have been proposed to explain PS-ED (Whyte and Mulsant, 2002). Additionally, several factors involving the severity of injury, cognitive impairment, pre-morbid depression, disability and localization of the stroke have been identified as predictors of PS-ED (Robinson, 1986; Hackett and Anderson, 2005; Ayerbe et al., 2013). However, some of these factors were inconsistent across studies. For example, earlier studies showed that left sided lesions that are close to the frontal lobe have been associated with depression (Robinson, 1986) whereas more recent studies showed no relation between the localization of stroke and depression after stroke (Carson et al., 2000).

Quantitative electroencephalography (qEEG) is a safe, cost-effective technique used to assess cortical activity and has been valuable in assessing emotion related networks. Among the qEEG parameters, frontal alpha power asymmetry has been especially of interest given its relation to emotional processes and pathological conditions such as major depressive disorder (MDD) and anxiety (Coan and Allen, 2004; Thibodeau et al., 2006; Harmon-Jones et al., 2010). Yet, it is unknown whether emotional disturbance secondary to other neurological conditions, such as stroke, is associated with similar EEG changes. In fact, qEEG has already been used in stroke as a predictive measurement for prognosis and clinical management in motor recovery (Finnigan and van Putten, 2013). However, use of qEEG in non-motor outcomes of stroke is limited (Schleiger et al., 2014) and to our knowledge there is no study assessing the qEEG correlates of post-stroke depression and anxiety.

Transcranial magnetic stimulation (TMS) is another technique that is useful in assessing cortical activity in both MDD and stroke. TMS studies assessing changes in cortical activity in patients with MDD have shown decreased excitability in the left hemisphere (Maeda et al., 2000; Fitzgerald et al., 2004), and decreased motor threshold in the right hemisphere (Bajbouj et al., 2006). In stroke, TMS studies demonstrated that inter-hemispheric asymmetry in cortical activity (Murase et al., 2004) is associated with functional recovery after stroke (Hendricks et al., 2002). Therefore, together with EEG, TMS could potentially help elucidate changes in cortical activity related to PS-ED.

In this cross-sectional preliminary analysis of 35 stroke subjects we investigated the associations between the emotion sub-domain of Stroke Impact Scale (SIS-Emotion) and several neurophysiologic measures obtained by EEG and TMS when adjusted for potential confounders such as age and time since stroke. Given that hemispheric asymmetry plays an important role in both stroke and mood disorders, we hypothesized that post-stroke changes in mood and emotional control is associated with inter-hemispheric imbalance that can be indexed by EEG and TMS.

## Methods

### Participants

This study analyzes the secondary data from 35 participants with chronic stroke who were initially enrolled in a larger clinical trial that compares different rehabilitation techniques. All subjects were over the age of 18 years, clinically stable and had clinical and neuro-imaging based diagnosis of stroke within 6–36 months prior to enrollment. The exclusion criteria were: (1) Mini-Mental Examination score lower than 24 or, for aphasic patients, inability to understand the rehabilitation tasks, (2) more than 1 stroke event, (3) psychoaffective disorders that prevented adherence to treatment and (4) joint damage and pain or deformities that makes the implementation of the therapy infeasible. Since the subjects were enrolled to participate in a trial assessing the effectiveness of rehabilitation techniques, some of these criteria were related to application of rehabilitation techniques. The study was approved by the Local Ethics Committee and a written consent was obtained from each subject.

### Stroke impact scale (SIS)

The Brazilian version of SIS 3.0 was used to measure quality of life and impact of stroke. For the purpose of this study we only analyzed the “emotion” sub-domain of SIS as a measure of mood and emotional control. SIS-Emotion consists of nine-items and has shown good criterion validity when compared to SF-36 Mental Health and Geriatric Depression Scale for SIS 2.0 (Duncan et al., 1999). Emotion sub-domain of SIS 3.0 has also shown good correlation with the Hospital Anxiety and Depression Scale (HADS; Carod-Artal et al., 2008; Vellone et al., 2014).

### Assessment of motor functions

We used the Fugl-Meyer (FM) assessment to test the association between motor impairment and SIS-Emotion. We also used FM to show that EEG and TMS findings of this study are specific to mood and emotional control.

### Transcranial magnetic stimulation (TMS)

TMS assessments included both single pulse and paired-pulse TMS protocols. We measured motor threshold (MT), intracortical inhibition (ICI), and intracortical facilitation (ICF) for both lesioned and unlesioned hemispheres. Motor threshold was defined as the minimum stimulus intensity necessary to elicit a motor-evoked potential (MEP) with the amplitude of at least a 50 μV in %50 of the trials. MEPs were recorded from first dorsal interosseous muscle (FDI). In the absence of MEPs, a maximum value of 100% was accepted as the MT. Paired-pulse protocol was used to measure ICI (ICI at 2 ms inter-stimulus interval) and facilitation (ICF at 10 ms inter-stimulus interval). In this protocol the conditioning pulse was set to 80% of MT while the test pulse was determined as the intensity eliciting an MEP of at least 1 mV. ICI and ICF were calculated as the ratio between the amplitude of MEP elicited during single pulse and during inhibition or facilitation.

### Electroencephalography (EEG)

EEG was recorded by using a 128-channel EEG cap with active electrodes (Acti-Champs, PyCorder, Brainvision LLC®) and linked-ear reference for 20 min. During recordings, subjects were asked to close their eyes in a resting position and instructed not to fall asleep. EEG sessions were monitored online for the effects of drowsiness and potential movements. The EEG data was analyzed offline with EEGLab and MATLAB (MATLAB R2012a, The MathWorks Inc. Natick, MA, 2000). To ensure EEG data was not contaminated by the effects of drowsiness only the first 6 min of EEG recordings were included in the analyses. The data was filtered automatically (high-pass at 1 Hz and low-pass at 35 Hz) and then cleaned from artifacts manually by an evaluator blinded to assessments.

#### Power asymmetry

**Figure 1:**
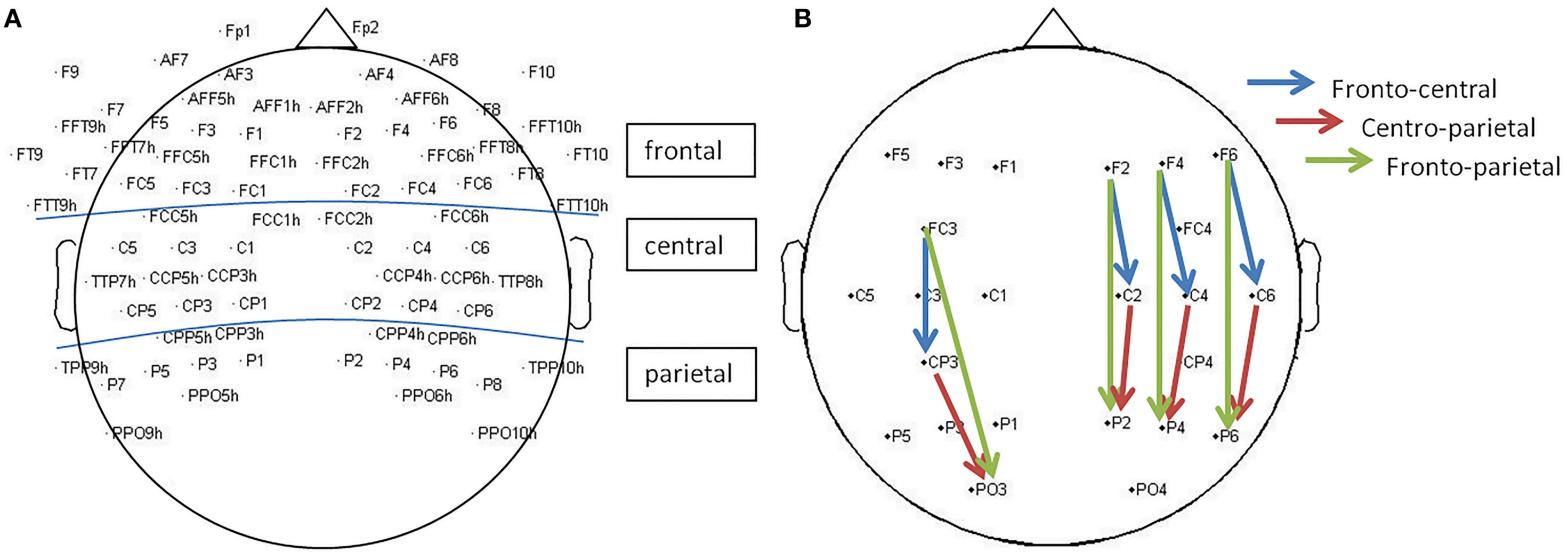
**Selected electrodes for EEG analysis**. **(A)** Analysis of inter-hemispheric power asymmetry and coherence: 40 pairs of electrode representing frontal (20 pairs), central (11 pairs), and parietal (9 pairs) areas were included. **(B)** Analysis of intra-hemispheric coherence. For each hemispheric side, 12 electrodes were chosen representing fronto-central (4 pairs), centro-parietal (4 pairs), and fronto-parietal (4 pairs) connections.

Power was calculated using fast Fourier transform (FFT; average of 5-s epochs with a 50% overlap) and averaged for the following EEG bands: theta (4–8 Hz), alpha (8–13 Hz) [including the sub-bands low-alpha (8–10 Hz) and high-alpha (1–13 Hz)], and beta [low-beta (13–20 Hz) and high-beta (21–30 Hz)]. Power asymmetry was determined by calculating the difference in power between the left hemisphere (LH) and right hemisphere (RH; e.g., L_alpha_ − R_alpha_) for 40 pairs of electrodes representing different cortical localizations: frontal (20 pairs), central (11 pairs), and parietal (9 pairs) (Figure 1). A positive asymmetry index represents greater left than right hemisphere EEG power, whereas a negative asymmetry index would suggest higher power in the right hemisphere.

#### Coherence

Coherence was calculated by using a MATLAB function -mscohere- that uses Welch's averaged modified periodogram to calculate magnitude squared coherence estimate. A coherence value between 0 and 1 was calculated for each frequency point and for each electrode pair (40 pairs for inter-hemispheric coherence and 24 pairs for intra-hemispheric coherence; Figure 1). We then averaged these values over certain frequency bandwidths including theta (4–8 Hz), alpha (8–13 Hz) [including the sub-bands low-alpha (8–10 Hz), high-alpha (10–13 Hz)], and beta [low-beta (13–20 Hz) and high-beta (20–30 Hz)].

### Statistical analysis

Statistical analyses were completed using STATA 12.1® (StataCorp LP, Texas) and MATLAB.

#### Covariance mapping—EEG data

In order to explore the correlation between SIS-Emotion and EEG data, we used a method introduced by Koening et al. which combines covariance analysis and resampling methods (“TANCOVA”) to overcome the issue of multiple testing across EEG electrodes (Koenig et al., 2008). To implement this technique into our analyses, we treated the data from each pair of electrode (left vs. right hemisphere power difference and coherence between each pair) as a single EEG “channel”. Initially all selected electrode pairs (40 pairs for inter-hemispheric power asymmetry and inter-hemispheric coherence, as well as 24 pairs for intra-hemispheric coherence) were included in the analysis. Then, we run the same analysis separately within each cortical region: frontal (20 pairs), central (11 pairs), parietal (9 pairs), left hemisphere (12 pairs), and right hemisphere (12 pairs). The main aim of this analysis was to preliminarily explore the association between EEG data and SIS-Emotion by including as many electrodes as possible while eliminating the problems of multiple testing. Following this analysis, we averaged the data over each cortical region (frontal, central, and parietal) in order to simplify the analyses in next steps.

#### Univariate analyses

Multiple univariate linear regression analyses were conducted to assess associations between the emotion sub-domain of SIS, SIS-Emotion (as the dependent variable), and each neurophysiologic, demographic, and clinical variable (as an independent variable). Neurophysiologic variables included EEG (i.e., frontal alpha power asymmetry, central alpha coherence) and TMS data (i.e., MT in the lesioned hemisphere). Demographic and clinical variables were age, gender, time since stroke, medication use, lesioned side, and Fugl-Meyer.

#### Confounders and multivariate models

In order to assess and adjust for possible confounders for EEG and TMS models (including the ones that were not significant in the univariate analysis), we added each demographic and clinical variable as an independent variable in multivariate regression models where SIS-Emotion was the dependent variable and each EEG and TMS variable is the main predictor. Each possible confounding variable was tested one at a time and when led to a more than 10% change in the β-coefficient of the main predictor, the variable was kept in the model. Finally, we performed multivariate regression analyses by forcing all significant confounders into final EEG and TMS models. *P* < 0.05 was accepted as significant.

#### Analysis testing whether significant neurophysiological findings are associated with motor outcomes

In order to assess the specificity of our findings we also tested whether the significant EEG and TMS variables (independent variables) are also predictors of motor (Fugl-Meyer) function (dependent variable). The aim was o show that these variables are not associated with motor disability.

#### Sensitivity analysis

In order to control and check whether individual values were driving our final results, we excluded outliers and repeated the analysis for the univariate and multivariate models. For each variable values that were above or below three inter-quartile range (Q1− 3IQR or Q3+ 3IQR) were defined as outliers and excluded.

#### Effect of lesion side on neurophysiologic and clinical parameters

In addition to the above analysis we compared patients with left and right hemisphere damage with regards to neurophysiological and clinical parameters using linear regression in order to further explore the effect of lesion side on these parameters.

## Results

Demographics and baseline characteristics of subjects are described in Table 1. Motor threshold for the lesioned hemisphere could not be obtained from three participants.

**Table 1:** **Demographics and baseline characteristics**.

	
**Gender (%)**	
Female	42.86
Male	57.14
**Age** (mean ± SD)	62 ± 13
**Lesion (%)**	
Cortical	65.71
Subcortical	28.57
Brain stem	5.71
**Hemispheric Side (%)**	
Right	54.29
Left	45.71
**Medications (%)**	
Antidepressant	25.71
Neuroleptic	2.86
Anticonvulsant	14.29
Benzodiazepine	5.71
**Time since stroke**	
(Months, mean ± SD)	15.3 ± 8.6

We divided our results into three sections in order to facilitate easy reading: A. Initial analyses of covariance mapping, univariate models and confounders as prerequirements of multivariate analyses; B. Multivariate models; C. Sensitivity Analysis (Sensitivity analysis presents the results in A and B but without outliers); D. Summary of the final multivariate models as also discussed in discussion; E. Other exploratory analyses.

### A. initial analyses of covariance mapping, univariate models, and confounders as prerequirements of multivariate analyses

With regards to results of covariance mapping (Table 2), SIS-Emotion significantly correlated with overall power asymmetry in low-beta band regardless of the region. Additional analysis of specific cortical regions revealed that power asymmetry (all frequency bands) and inter-hemispheric coherence (alpha band) over frontal areas as well as intra-hemispheric coherence (beta band) within the left hemisphere are significantly associated with post-stroke mood and emotional control.

**Table 2:** **Results for covariance mapping**.

	**Theta *p*-values**	**Alpha *p*-values**	**Low-alpha *p*-values**	**High-alpha *p*-values**	**Low-beta *p*-values**	**High-beta *p*-values**
**POWER ASYMMETRY**						
All regions^**^	0.056	0.186	0.249	0.150	**0.030**	**0.024**
Frontal^*^	**0.026**	**0.026**	**0.027**	**0.030**	**0.028**	**0.030**
Central^*^	0.879	0.568	0.380	0.874	0.906	0.827
Parietal^*^	0.093	0.749	0.781	0.509	0.306	0.556
**INTER-HEMISPHERIC COHERENCE**						
All regions^**^	0.359	0.121	0.109	0.137	0.119	0.174
Frontal^*^	0.174	**0.044**	**0.031**	0.073	0.068	0.133
Central^*^	0.530	0.199	0.164	0.263	0.340	0.319
Parietal^*^	0.244	0.110	0.281	0.055	0.054	0.103
**INTRA-HEMISPHERIC COHERENCE**						
All regions^**^	0.489	0.732	0.753	0.533	0.753	0.197
Left^*^	0.139	0.511	0.6104	0.369	**0.039**	**0.011**
Right^*^	0.814	0.698	0.600	0.861	0.926	0.941

*Showing the p-values for the correlation between SIS-Emotion and EEG data*.

** *Analysis for all regions included 40 pairs of electrodes for power asymmetry and inter-hemispheric coherence, and 24 pairs of electrodes for intra-hemispheric coherence*.

* *Electrodes were further grouped into different cortical regions in order to explore the association between SIS-Emotion and cortical activity in these specific brain regions: frontal (20 pairs), central (11 pairs), parietal (9 pairs), left (12 pairs), right (12 pairs). P < 0.05 are shown in bold*.

Similar results were also found for the univariate linear regression models which showed significant associations between the dependent variable SIS-Emotion, and (1) alpha power asymmetry, (2) alpha coherence, and (3) MT in the unlesioned hemisphere.

We also found that age is a significant predictor for SIS-Emotion (Table 3) and is a common confounder (change in β-coefficent more than 10%) for the association between the dependent variable SIS-Emotion, and multiple EEG and TMS variables. Therefore, it was forced into all final multivariate models. Other important confounders were included only for the models that they were confounder for. Models became significant or remained significant discussed below (B. Multivariate models).

**Table 3:** **Main effects of possible confounders**.

	***p*-value**	**β-coeff**
Medication use	0.871	−1.13
Time since stroke	0.398	−0.32
Motor function-FuglMeyer	0.463	0.30
Lesioned hemisphere	0.574	3.64
Gender	0.701	−2.5
Age	**0.016**	−0.56

*Results of univariate analyses for the dependent variable SIS-Emotion, showing p-values and β-coefficients for possible confounders. P < 0.05 are shown in bold*.

### B. multivariate models

#### Multivariate models-EEG variables with confounders

##### Power asymmetry

We found that SIS-Emotion was significantly associated with beta power asymmetry (low-beta: *p* = 0.005, β = 180.52, Adj-*R*^2^ = 0.30) in frontal regions and alpha power asymmetry (low-alpha: *p* = 0.040, β = 10.67, Adj-*R*^2^ = 0.25) in parietal regions when adjusted for age and time since stroke (only for parietal low-alpha).

##### EEG coherence

In addition to power asymmetry, SIS-Emotion was significantly associated with lower functional connectivity measured by inter-hemispheric EEG coherence in *alpha band* over frontal (low-alpha: *p* = 0.042, β = 54.63, Adj-*R*^2^ = 0.22), central (alpha: *p* = 0.040, β = 88.14, Adj-*R*^2^ = 0.22, low-alpha: *p* = 0.031, β = 66.43, Adj-*R*^2^ = 0.22) and parietal areas (alpha: *p* = 0.037, β = 54.35, Adj-*R*^2^ = 0.21) as well as in *beta band* over parietal areas (low-beta: *p* = 0.029, β = 57.51, Adj-*R*^2^ = 0.24). These results were adjusted for age, side of lesion (only for parietal alpha and central low-alpha coherence), time since stroke (only for parietal low-beta), and gender (only for parietal low-beta). No other significant association was found for power and coherence in other frequency bands and locations (*p* > 0.05).

##### Multivariate models-TMS variables with confounders

A significant association was found between MT in the unlesioned hemisphere and SIS-Emotion (*p* = 0.003, β = −0.73, Adj-*R*^2^ = 0.32) when adjusted for age and gender. ICF in the unlesioned hemisphere (adjusted for age) was also significantly associated with SIS-Emotion (*p* = 0.043, β = −5.15, Adj-*R*^2^ = 0.22). There was no association between SIS-Emotion and other TMS variables.

### C. sensitivity analysis

*P*-values for the univariate models after exclusion of outliers are shown in Table 4.

**Table 4A:** **Univariate analysis-EEG**.

	**Theta**		**Alpha**		**Low-alpha**		**High-alpha**		**Low-beta**		**High-beta**	
	***p*-value**	**β-coeff**	***p*-value**	**β-coeff**	***p*-value**	**β-coeff**	***p*-value**	**β-coeff**	***p*-value**	**β-coeff**	***p*-value**	**β-coeff**
**POWER ASYMMETRY**												
Frontal^*^	0.433	43.9	**0.002**	170.6	0.176	50.09	0.327	88.8	0.068	242.1	0.067	290.2
Central^*^	**0.028**	101.5	0.470	12.5	0.226	−29	**0.002**	−144.3	0.285	−144.5	0.845	70.9
Parietal^*^	0.929	3.4	0.915	3.9	0.175	29.19	0.710	−15.91	0.327	−160.5	0.670	64.9
Occipital^*^	0.220	−47.3	0.473	−42.3	0.712	13.8	0.291	−163.8	0.580	−114.0	0.878	55.4
**INTER**−**HEMISPHERIC COHERENCE**												
Frontal	0.369	30.2	**0.024**	79.4	**0.020**	65.3	0.073	71.4	0.200	59.3	0.240	43.8
Central	0.324	37.9	**0.006**	114.0	**0.021**	71.5	**0.026**	92.9	0.237	56.2	0.332	29.3
Parietal	0.686	15.8	0.089	79.7	0.292	40.8	0.118	55.1	0.306	41.6	0.215	32.0
Occipital	0.634	7.6	0.598	9.6	0.776	5.2	0.473	12.1	0.638	7.8	0.990	−0.2
**INTRA-HEMISPHERIC COHERENCE**												
**Left Hemisphere**												
Fronto-Central	0.658	8.7	0.676	8.9	0.439	15.4	0.991	0.2	0.986	−0.4	0.876	3.1
Centro-Parietal	0.328	23.9	0.391	23.0	0.327	22.6	0.572	14.7	0.624	14.3	0.404	19.8
Fonto-Parietal	0.169	46.0	0.268	38.9	0.160	36.4	0.717	14.0	0.756	−14.0	0.966	−1.2
**Right Hemisphere**
Fronto-Central	0.191	32.4	0.052	54.8	0.098	38.2	**0.038**	65.5	0.337	32.5	0.208	36.03
Centro-Parietal	0.782	7.0	0.610	11.6	0.734	7.2	0.545	12.7	0.725	10.2	0.462	17.6
Fonto-Parietal	0.526	25.2	0.227	41.8	0.394	21.5	0.187	48.6	0.852	−9.8	0.319	41.4

*Results of univariate linear regression analyses for the dependent variable SIS-Emotion; showing p-values and β-coefficients for each EEG variable after outliers were excluded. P < 0.05 are shown in bold*.

**Table 4B:** **Univariate analysis-TMS**.

	**TMS**	
	***p*-value**	**β-coeff**
**ICF**		
Lesioned	0.760	−1.45
Unlesioned	0.104	−4.48
**ICI**		
Lesioned	0.510	−5.54
Unlesioned	0.882	1.22
**MT**		
Lesioned	0.964	−0.01
Unlesioned	**0.022**	−0.54

*Results of univariate linear regression analyses for the dependent variable SIS-Emotion; showing p-values and β-coefficients for each TMS variable after outliers were excluded. P < 0.05 are shown in bold*.

**Figure 2:**
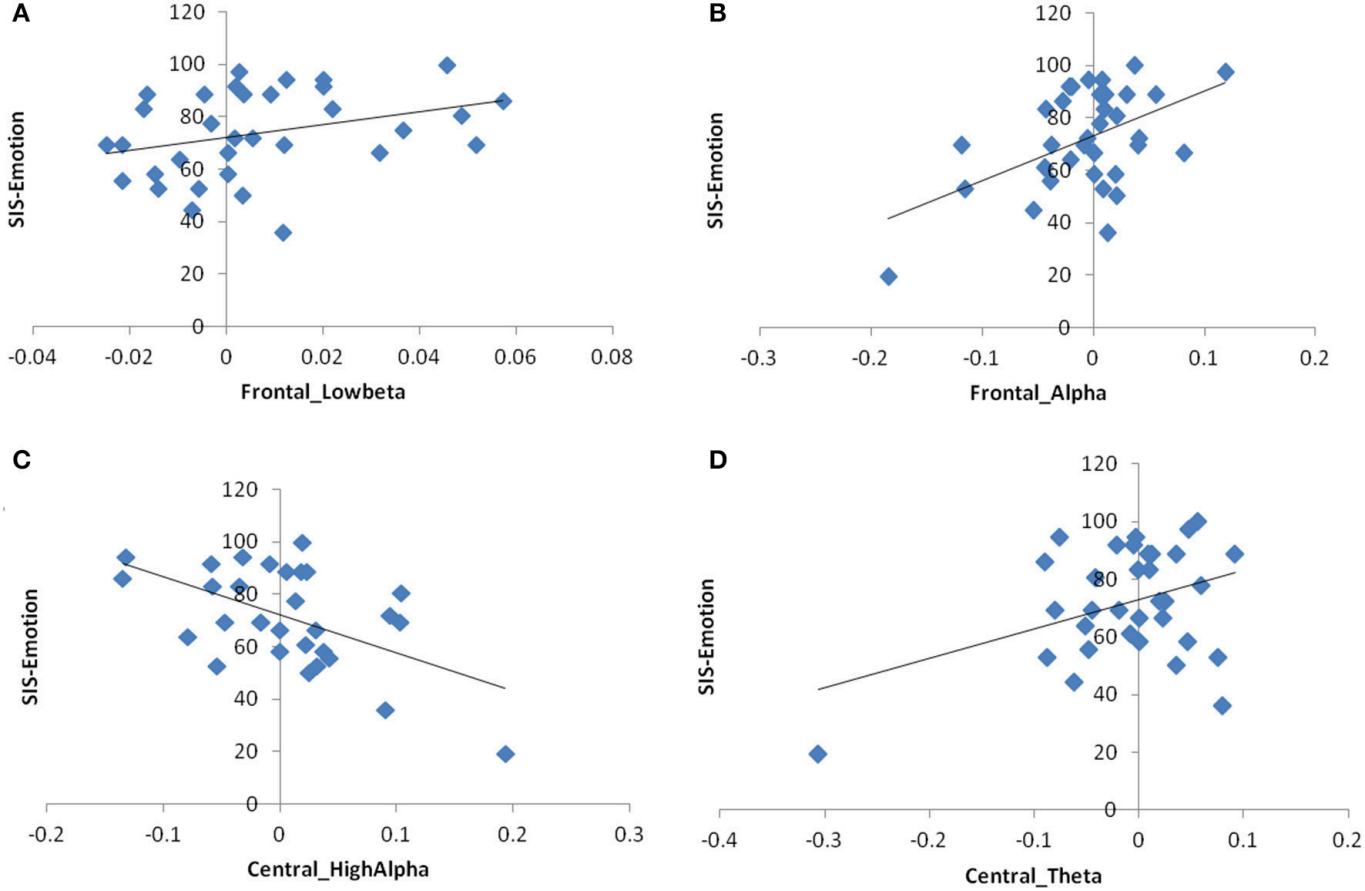
**Scatter plot graphs for the relationship between SIS-Emotion and EEG power asymmetry as measured by the difference between left and right hemisphere**. **(A)** Frontal low-beta power asymmetry, **(B)** Frontal alpha power asymmetry, **(C)** Central high-alpha power asymmetry, **(D)** Central theta power asymmetry.

In the multivariate models without outliers, *alpha power asymmetry* in frontal (*p* = 0.008, β = 145.02, Adj-*R*^2^ = 0.29) and central (only high-alpha, *p* = 0.006, β = −133.2, Adj-*R*^2^ = 0.27) regions, as well as *theta asymmetry* in central areas (*p* = 0.025, β = 95.86, Adj-*R*^2^ = 0.25) became significant for the dependent variable SIS-Emotion when adjusted for age. In beta band, the main effect of power asymmetry in the sub-band low-beta remained significant (*p* = 0.045, β = 247.33, Adj-*R*^2^ = 0.21; Figure 2). On the other hand alpha asymmetry over parietal areas became not significant.

**Figure 3:**
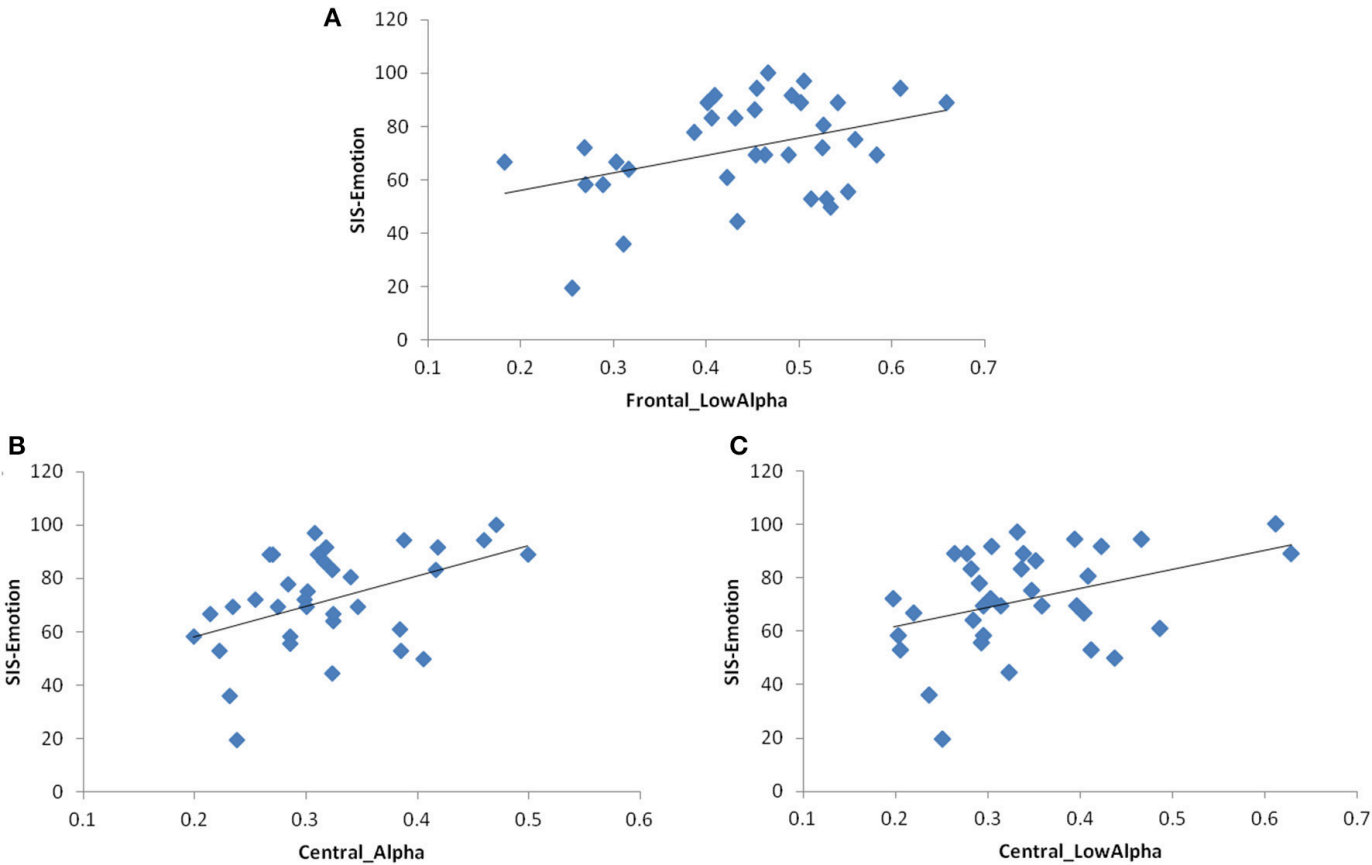
**Scatter plot graphs for the relationship between SIS-Emotion and inter-hemispheric EEG coherence**. **(A)** Frontal low-alpha coherence, **(B)** Central alpha coherence, **(C)** Central low-alpha coherence.

**Figure 4:**
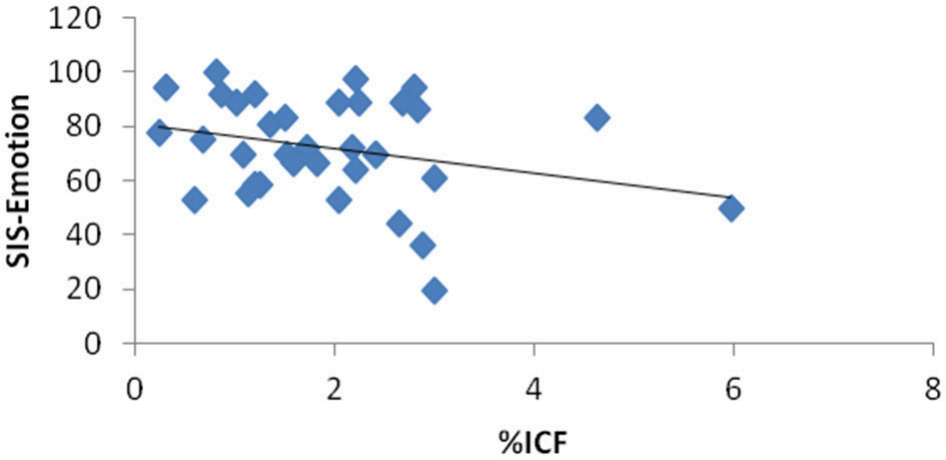
**Scatter plot graph for the relationship between SIS-Emotion and intracortical facilitation in the unlesioned hemisphere measured by TMS**.

With regards to association between SIS-Emotion and EEG coherence, inter-hemispheric coherence over parietal areas (alpha and beta) became not significant when outliers were excluded. The models for frontal and central regions remained same as there were no outliers for these regions (Figure 3). Multivariate models with TMS variables also remained unchanged (Figure 4).

The results of the sensitivity analysis suggest that the association between SIS-Emotion and EEG variables is more robust for frontal and central areas as compared to parietal areas which seemed to be driven by outliers. Therefore, in our discussion we focused on the results without outliers.

### D. summary of the final multivariate models

In summary, subjects with right greater than left hemisphere EEG power in beta and alpha band over frontal areas as well as in theta band over central areas were found to have more difficulty in post-stroke mood and emotional control (lower SIS-Emotion scores). Interestingly, this relation seems to be inverted for alpha power asymmetry over central areas. The relation between SIS-Emotion and EEG coherence was in line with the results for power asymmetry, and suggests that reduced functional connectivity in alpha band over frontal and central areas is also associated with difficulty in post-stroke mood and emotional control. In addition to EEG variables, the multivariate models with TMS variables showed that MT and ICF in the unlesioned hemisphere could be relevant markers for mood and emotional control after stroke.

### E. other exploratory analyses

#### Analysis testing whether significant neurophysiological findings are associated with motor outcomes

We tested whether the significant EEG and TMS variables from this study are also correlated with motor function outcomes (Fugl-Meyer). In these models Fugl-Meyer was the dependent variable and each EEG and TMS variable was an independent variable. None of the EEG and TMS variables that were significant for SIS-Emotion were associated with Fugl-Meyer (also see the data from Simis et al., 2015).

#### Effect of lesion side on neurophysiologic and clinical parameters

There was a main effect of the lesion side for the following dependent EEG variables: (1) frontal beta power asymmetry (high-beta: *p* = 0.037, β = 0.03, Adj-*R*^2^ = 0.09), (2) parietal inter-hemispheric alpha coherence (low-alpha: *p* = 0.037, β = −0.09, Adj-*R*^2^ = 0.09), and (3) left centro-parietal (low-alpha: *p* = 0.019, β = −0.11, Adj-*R*^2^ = 0.13) and fronto-parietal (low-alpha: *p* = 0.049 β = −0.08, Adj-*R*^2^ = 0.09) intra-hemispheric alpha coherence.

## Discussion

In this study we found significant associations between EEG/ TMS measures and post-stroke mood and emotional control in patients with chronic stroke. These results indicate that patients who report having more depressive symptoms and anxiety also have (1) higher inter-hemispheric imbalance in cortical activity as measured by EEG power and coherence and (2) higher intracortical excitability and motor threshold in the unlesioned hemisphere as measured by TMS when adjusted for confounders. Furthermore, to our knowledge this is the first study that emphasizes the potential use of EEG and TMS to index neurophysiologic changes associated with post-stroke mood and emotional control.

With regards to the EEG findings in beta band, we found that post-stroke mood and emotional control is associated with greater frontal beta power asymmetry. The direction of the asymmetry index indicates that subjects with relatively greater right than left hemisphere beta power have more difficulties in mood and emotional control. This finding is consistent with previous studies showing higher EEG beta power in the right frontal regions in depressive disorders (Pizzagalli et al., 2002; Volf and Passynkova, 2002; Flor-Henry et al., 2004). Beta oscillations, are inhibition based rhythms that are thought to be produced by GABAergic potentials in inhibitory interneurons and pyramidal cells (Faulkner et al., 1999; Whittington et al., 2000) and are associated with motor control, arousal and attention (Merica et al., 1998; Uhlhaas et al., 2008). They are thought to reflect hyperactive neural circuits (Traub et al., 1999) and increased metabolic activity (Cook et al., 1998). Therefore, increased beta power in the right hemisphere may suggest hyperactivity in this region (Flor-Henry et al., 2004). In fact, right frontal hyperactivity has been associated with negative affect and withdrawal (Sutton and Davidson, 1997) as well as the presence of melancholia and anxiety in depressed individuals (Pizzagalli et al., 2002). Moreover, beta oscillations have been suggested as potential marker for plasticity (Rossiter et al., 2014). Therefore, beta oscillation may also reflect post-stroke plastic changes in the networks related to arousal and attention.

In addition to beta band, we also found that post-stroke mood and emotional control is associated with power asymmetry and reduced connectivity in alpha band over frontal and central areas.

Given the inverse relation between alpha power and cortical activity, the direction of the power asymmetry index over frontal regions suggest hypoactivity in the right frontal cortex or hyperactivity in the left frontal cortex, or both. At first this seems to contradict with not only one of the common EEG findings in depression and anxiety (right prefrontal hyperactivity), but also our findings regarding frontal beta asymmetry (which also suggest hyperactivity in the right frontal cortex). However, it is possible that greater activity in the left frontal cortex as measured by reduced alpha power represents different neural interactions and has other implications. For example several authors suggested that greater left prefrontal hyperactivity is involved in anxious apprehension while right sided hyperactivity is related to anxious arousal (Heller et al., 1997; Engels et al., 2007). Indeed it is not uncommon that patients with stroke present with both anxious arousal and apprehension (Mukherjee et al., 2006) which could explain the concurrent hyperactivity in different regions of frontal areas.

The relation between SIS-Emotion and central power asymmetry in high-alpha band was in the opposite direction of what was observed between SIS-Emotion and frontal alpha asymmetry, with patients who have greater left than right alpha power (hypoactivity in the left or hyperactivity in the right) experiencing more difficulty in mood and emotional control. In depression, posterior hyperactivation in right parieto-temporal cortex has been associated with the presence of co-morbid anxiety (Bruder et al., 1997). Even though we did not find any significant correlation over parietal regions in our final models, hyperactivation in the right central areas may also indicate presence of anxiety in stroke patients. Different topological distribution of power asymmetry (central vs. parietal) may be due to methodological differences (for example we did not group the electrodes for parieto-temporal cortex separately) or due to the shift in brain activity as a result of cortical re-organization after stroke. Another possibility is that, together with theta asymmetry (which was in the opposite direction of alpha asymmetry), alpha asymmetry over central regions represents secondary cognitive impairment in stroke patients. Alpha and theta power are inversely related when they are used to index cognition and memory, and the presence of large power in high-alpha band in addition to small power in theta band is thought to indicate better cognitive performance (Klimesch, 1999). Even though it is not possible to conclude any association without the assessment of cognitive functions, knowing that cognitive impairment is common in stroke and can directly result from depression and anxiety (Mukherjee et al., 2006), one can argue that there might be overlapping EEG findings related to cognition and mood.

Consistent with our findings in power asymmetry we also found that post-stroke mood and emotional control was associated with reduced functional connectivity in alpha band over frontal and central areas. Studies investigating functional connectivity in MDD revealed mixed results (Veer et al., 2010; Zhou et al., 2010; Olbrich and Arns, 2013). Yet, our results are close to Knott et al. (2001) who found that depressive patients have reduced inter-hemispheric coherence in delta, theta, alpha, and beta bands of EEG in all anterior and posterior homologous pairs of channels (Knott et al., 2001). Pathological conditions affecting the integrity of the neural tissue will have structural and functional consequences in the area where the insult occurred. It can be assumed that reduction in inter-hemispheric connectivity reflect anatomical, adaptive, and maladaptive changes to neural connections between the lesioned and unlesioned hemisphere following stroke (Kukke et al., 2015).

One possible explanation for our EEG findings is the “depression network model” that identifies the depressed state as a dysfunction of a “network” rather than single brain region including adaptive and maladaptive compensatory processes. It is possible that in stroke secondary maladaptive changes in remote areas result in changes in the “depression network” which involves connections among neocortex, cingulate, limbic system, striatum, and thalamus (Mayberg, 1997, 2003). In fact, recent neuroimaging studies in stroke confirmed changes to the resting-state networks including the default-mode network (DMN; Wang et al., 2014; Thiel and Vahdat, 2015). Concurring with the “depression network” model, alterations in functional connectivity within DMN are also known to be related to depression (Mayberg, 1997; Greicius et al., 2007; Dutta et al., 2014). Since alpha oscillations (8–13 Hz) are the main regulator for DMN (Knyazev et al., 2011), it is likely that any disruption in the DMN or depression network, such as in stroke, could present as changes in EEG alpha oscillations and synchronization/desynchronization of the connectivity in alpha band.

The observed qEEG findings and its relationship with measurements of mood and emotions offers the possibility to be used as markers for treatment by EEG biofeedback entrainment, as it has been shown to be useful in stroke and memory impairment (Nelson, 2007), information processing (Lee et al., 2015), and motor function rehabilitation (Yilmaz et al., 2014).

We also found that higher ICF and higher motor threshold (MT) in the unlesioned hemisphere are associated with lower (worse) SIS-Emotion scores. TMS is advantageous over EEG considering that it can evaluate the cortical activity within each hemisphere, whereas EEG indices such as power asymmetry and coherence are difficult to interpret regarding which hemisphere mostly contributes to the asymmetry or reduced connectivity. Given that both ICF and EEG beta oscillations reflect interactions between glutamergic excitatory and GABAergic inhibitory neurons (Whittington et al., 2000; Paulus et al., 2008), increased ICF in the unlesioned hemisphere together with decreased inter-hemispheric coherence in beta band might represent the shift in the cortical activity toward unlesioned hemisphere. In stroke, persistent disinhibition in the unlesioned hemisphere has been associated with maladaptive plasticity and motor recovery (Manganotti et al., 2008) and it is possible that same maladaptive changes are related to post-stroke mood and emotional control. However, hyperactivity in the unlesioned hemisphere alone is not sufficient to explain the findings in EEG power asymmetry. One explanation is that both the plastic changes in motor areas and secondary disruption to “depression network” contribute to post-stroke mood and emotional control. In addition to the ICF, we found that MT in the unlesioned hemisphere is also associated with lower scores on SIS-Emotion. Even though this seems to contradict with the relationship between ICF and SIS-Emotion at first; when compared to ICF, MT does not provide information on intracortical connections. Also, while MT in the lesioned hemisphere strongly correlates with motor recovery, it seems to be inadequate to assess the functional changes in the unlesioned hemisphere (Stinear et al., 2015; Simis et al., 2016). Therefore, it is unlikely that the relationship between MT in the unlesioned hemisphere and SIS-Emotion is truly accounted for by maladaptive changes in the unlesioned hemisphere.

Even though there is conflicting evidence, one important factor implicated in post-stroke depression is the lesion side. Therefore, we further compared the patients with left and right sided lesions with regards to neurophysiologic and clinical parameters. We found that three variables were significantly associated with the lesion side: (1) frontal power asymmetry in high-beta band, (2) parietal power asymmetry in low-alpha band, and (3) intra-hemispheric coherence in alpha band within the left hemisphere. These results suggest that patients have relatively greater frontal high-beta power in the lesioned side. Also patients with left sided injuries have reduced connectivity within the left hemisphere as well as in between the two hemispheres over parietal areas as compared to patients with right sided injuries. Even though there was no direct relation between SIS-Emotion and these variables, it seems that the lesion side might have certain effects on brain activity regardless of the change in mood and emotional control.

Altogether, our results support our initial hypothesis suggesting the association between inter-hemispheric imbalance and post-stroke mood and emotional control. Even though inter-hemispheric imbalance is a common finding in stroke and is associated with motor recovery (Murase et al., 2004; Simis et al., 2015), the lack of correlation between FM and variables that are associated with mood and emotion suggests that observed neurophysiologic changes are not related to motor impairment. Supporting this, we also did not find any association between FM and post-stroke mood and emotional control, and FM was not a confounder for any of the EEG and TMS variables. Indeed, in our recent study (Simis et al., 2015), we showed that EEG variables do not directly correlate with motor function but rather specify the association between motor threshold in the lesioned hemisphere and FM.

It is important to note the limitations of this study. First of all, even though multiple univariate linear regression models were tested, no correction was made for multiple comparisons. On the other hand the results of our initial analysis using covariance mapping and resampling methods were in line with the results of our multivariate models suggesting that it is unlikely that the effects were due to chance. Secondly, it is important to note that measuring coherence based on scalp EEG channels can be confounded with several unwanted effects of volume conduction. Therefore, the results regarding EEG coherence should be interpreted cautiously. Another limitation of this study is the lack of control group. Future studies are needed to compare the findings in healthy subjects and in patients with affective disorders or anxiety only.

## Conclusion

To our knowledge this is the first study assessing neurophysiologic markers in post-stroke mood and emotional control as indexed by EEG and TMS. Our results suggest that difficulties in mood and emotional control after stroke is associated with greater inter-hemispheric imbalance in EEG power and coherence as well as increased excitability in the unlesioned hemisphere measured by TMS. These results are important for guiding future studies investigating the neurophysiologic mechanisms of post-stroke emotional disturbance, developing diagnostic algorithms, and assessing treatment response.

## Author contributions

MS, FF, MI, LB designed the study. MS and RA collected the data. DD, MS, and FF analyzed the data and wrote the first draft. All authors (DD, MS, MI, AB, LQ, RA, FF, and LB) interpreted the results and contributed to the final manuscript.

## Funding

This article was produced as part of the activities of FAPESP Center for Neuromathematics (grant #2013/07699-0, S. Paulo Research Foundation). The authors gratefully acknowledge NIH support (Support to FF—NIH grant 5 R21 HD079048-02).

### Conflict of interest statement

The authors declare that the research was conducted in the absence of any commercial or financial relationships that could be construed as a potential conflict of interest. The reviewer RP and handling Editor declared their shared affiliation, and the handling Editor states that the process nevertheless met the standards of a fair and objective review.
